# Genotype-Phenotype Correlation in Cri du Chat Patients Carrying One or Two Copies of the *TRIO* Gene

**DOI:** 10.3390/ijms27219400

**Published:** 2026-10-22

**Authors:** Andrea Guala, Fabio Tognon, Giuseppe Minniti, Fabio Sirchia, Silvia Kalantari, Michela Malacarne, Chiara Baldo, Alessandro Ferretti, Carla Liaci, Davide Politano, Roberto De Icco, Giorgio Roberto Merlo, Enza Maria Valente, Cesare Danesino

**Affiliations:** 1 Division of Paediatrics, Castelli Hospital, 28922 Verbania, Italy; gualaandrea0@gmail.com; 2 Scientific Committee of Associazione Bambini Cri du Chat (A.B.C.), 50026 Firenze, Italy; tognonf@gmail.com (F.T.); gminniti.981@gmail.com (G.M.); 3 Department of Molecular Medicine, University of Pavia, 27100 Pavia, Italy; fabio.sirchia@unipv.it (F.S.); silvia.kalantari@unipv.it (S.K.); 4 Medical Genetics Unit, IRCCS San Matteo Foundation, 27100 Pavia, Italy; 5 Laboratory of Human Genetics, IRCCS Istituto Giannina Gaslini, 16147 Genova, Italy; michelamalacarne@gaslini.org (M.M.); chiarabaldo@gaslini.org (C.B.); 6 Paediatrics Unit, Neuroscience, Mental Health and Sense Organs (NESMOS) Department, Faculty of Medicine and Psychology, Sapienza University of Roma, 00189 Roma, Italy; alessandro.ferretti@uniroma1.it; 7 Department of Stem Cell Biology and Regenerative Medicine, Eli and Edythe Broad CIRM Center for Regenerative Medicine and Stem Cell Research, Keck School of Medicine, University of Southern California, Los Angeles, CA 90033, USA; carlaliaci@gmail.com; 8 Child Neurology and Psychiatry Unit, IRCCS Mondino Foundation, 27100 Pavia, Italy; davide.politano@mondino.it; 9 Department of Brain and Behavior Neuroscience, University of Pavia, 27100 Pavia, Italy; roberto.deicco@mondino.it; 10 Department of Molecular Biotechnology and Science for Health, University of Torino, 10126 Torino, Italy; giorgioroberto.merlo@unito.it

**Keywords:** Cri du Chat syndrome, 5p-, *TRIO* gene, head circumference, neuro-developmental steps, gender

## Abstract

Cri du Chat syndrome (CdCS) results from a variable-sized deletion of the short arm of chromosome 5 (5p) and is characterized by marked phenotypic heterogeneity. Previous genotype–phenotype correlation studies have primarily focused on deletion size, an approach inherently limited by the large number of genes within the deletion. A gene-centered strategy may therefore offer a better framework for interpreting specific clinical features. Among candidate genes on 5p, *TRIO* plays a critical role in RAC1-mediated neurodevelopment; furthermore, in related neurodevelopmental disorders, loss-of-function variants are associated with microcephaly, whereas gain-of-function variants are associated with macrocephaly. Clinical, cytogenetic, and molecular data were retrieved from the main database of the Italian charity A.B.C. (Associazione Bambini Cri du Chat). Fifty-two patients, classified as carriers of one or two copies of the *TRIO* gene, were included based on the availability of head circumference (HC) measurements, developmental milestones (sitting, walking, and first words) data, and a CGH array. All data were analyzed in relation to *TRIO* allele dosage, deletion size, and sex. All patients showed a reduced HC compared with reference curves. However, HC measurements were consistently higher in patients carrying two *TRIO* alleles versus those carrying only one. Deletion size did not correlate with HC or developmental milestones. Patients with two *TRIO* alleles generally achieved motor and language milestones earlier, and some differences between males and females, suggested the possibility of sex-specific differences. These findings indicate that *TRIO* allele dosage might contribute to head size and developmental outcomes in CdCS independent of deletion size. A gene-centered approach could thus improve genotype–phenotype correlations in chromosomal disorders and highlight the importance of considering sex-specific effects in clinical and rehabilitative management.

## 1. Introduction

Cri du Chat syndrome (CdCS), first described in 1963 by Jerome Lejeune [1], is caused by a deletion of variable size on the short arm of chromosome 5. Following improvements in molecular diagnostic techniques, deletion sizes have been studied in detail, and attempts to establish genotype-phenotype correlations (GPCs) between specific deleted regions and clinical symptoms—particularly microcephaly and a high-pitched cry—have been reported [2,3].

A possible limitation of these GPCs is that the deleted regions vary in size and often encompass tens or even hundreds of genes. Many of these genes have diverse and often unknown functions, making their contribution to the CdCS phenotype difficult to assess. The extensive phenotypic variability of CdCS has recently been addressed [3]; specifically, the authors proposed that when deletion size alone is insufficient to explain a GPC, the specific chromosomal location of the deletion and its gene content may provide an alternative explanation.

In their study, the authors also evaluated whether the most frequent findings might be interrelated. Using Kendall’s tau-b analysis, their results showed that microcephaly (observed in more than 65% of cases and found to be more common in males at 84.3%) correlated with epicanthus, a narrow nasal bridge, and ear alterations. Furthermore, and highly relevant to the present study, deletion size has been proposed as an indicator of microcephaly [3,4].

These previous findings prompted us to further examine these GPCs by adopting a gene-centered analysis, as we believe that a gap still exists between GPCs related to deletion size and the recognizable effects of single-gene loss. Our study proposes a shift toward gene-level resolution studies.

From the main database (mdb) of the Italian charity A.B.C. (Associazione Bambini Cri du Chat), we collected available data on head circumference (HC) and other developmental milestones, including the ages at which each patient was able to sit independently, walk independently, and speak their first words, as well as whether these developmental milestones had not been reached at the time the clinical data were recorded.

These data were compared between two groups of cases classified as: (i) carriers of 5p deletions including the Triple Functional Domain (*TRIO*) gene (thus carrying only one allele on the structurally normal chromosome) and (ii) carriers of deletions not including the *TRIO* gene (two alleles present). The choice of the *TRIO* gene was motivated by previous studies: *TRIO* is located at the end of 5p12, is expressed starting from mid-late fetal stages in the human cortex [5], and codes for a positive regulator of Rho-GTPases. These, in turn, are transducing enzymes that control the dynamics of the neuronal cytoskeleton.

*TRIO* heterozygous mutations cause two genetic neurodevelopmental disorders: MRD44 (OMIM #617061) and MRD63 (OMIM #618825). The phenotypic heterogeneity of TRIOpathies depends on the domain affected: patients harboring mutations that alter the activity of the GEFD1 domain, or that lead to a nonfunctional protein (loss of function), display developmental delay, microcephaly, and intellectual disability, while mutations in the spectrin domain are associated with macrocephaly [6,7,8,9]. CdC patients with the loss of one *TRIO* allele are expected to be equivalent to those carrying a typical loss-of-function mutation in *TRIO*. Notably, the disruption of *Trio* in mice mimics the phenotype caused by loss-of-function mutations in the GEFD1 domain, resulting in reduced brain size [10,11].

The aim of this study is to investigate a GPC in CdCS patients between the presence of one or two copies of the *TRIO* gene, microcephaly and the aforementioned developmental milestones. The working hypothesis is that *TRIO* haploinsufficiency independently drives specific developmental outcomes and impacts HC size.

## 2. Results

### 2.1. The Patients

We collected clinical, cytogenetic, and molecular data from the main database of families registered with the Italian charity A.B.C. (Associazione Bambini Cri du Chat). From an initial retrospective cohort of 258 patients diagnosed with CdCS (carrying different 5p deletions), a well-characterized subset of 52 cases with available array-CGH genomic data was isolated for further analysis. While this subset represents the core cohort for genetic stratification, missing clinical metrics (such as HC or specific developmental milestones) were handled via pairwise exclusion in their respective sub-analyses. Patients were initially stratified based on their *TRIO* gene status, yielding 13 *TRIO*+ cases (gene preserved) and 39 *TRIO*− cases (gene deleted).

Longitudinal milestone data were sequentially evaluated for each subgroup. For the *TRIO*+ subgroup (n = 13), full data availability was maintained for both sitting and walking milestones, while one case (ID 51) was excluded from the final speech analysis due to missing data (leaving n = 12 complete cases). Within the *TRIO*− subgroup (n = 39), 2 cases (ID 86 and ID 137) lacked sitting milestone data and were excluded at the first developmental node. Of the remaining 37 cases evaluated for independent walking, an additional two cases (ID 100 and ID 257) were excluded due to missing values. At the final speech milestone node, three further cases (ID 75, ID 95, and ID 89) were excluded, resulting in a final dataset of 32 *TRIO*− cases with complete longitudinal developmental metrics. The details of this selection cascade are presented in the flow chart in Figure S1 in Supplementary Information (SI).

The mdb also includes some cases previously reported [12]; as this family attended A.B.C. meetings, their data were subsequently incorporated into the database. For the present analysis, we selected from the mdb all cases (n = 52) for whom genetic data (CGH array) were available. HC measurements (available for 48 out of 52 cases), either at birth (SI Table S1) or later in life (SI Table S2) and clinical data (specifically, the age at which the patient achieved sitting alone, walking alone, and speech) were then added as part of the longitudinal tracking (SI Table S1 and SI Figure S1).

This subgroup comprised 28 females (53.85%) and 24 males (46.15%) and is detailed in SI Table S1, which contains the following patient information: ID, sex, number of TRIO alleles, pregnancy duration in weeks, HC measures at birth, distal and proximal breakpoints, deletion size, and the ages in months at which the developmental milestones studied (sitting alone, walking alone, and speech) were achieved. Throughout the manuscript, patients are identified by their “ID” followed by the corresponding number in the list; ID 232, ID 233, and ID 234 correspond to previously published cases [12].

For each patient, a caregiver signed an informed consent form for the inclusion of data in the database and for scientific use. The prenatal HC percentile curves, corresponding to the 98th, 50th, and 2nd centiles, were derived from Papageorghiou et al. [13]. The postnatal HC percentile curves, corresponding to the 98th, 50th, and 2nd centiles in CdCS patients, are those reported by Marinescu et al. [14]. HC measurements were obtained at different ages across patients, who had variable gestational ages at birth (SI Table S2). To compare HC, we used Gompertz curves [15]. The ages at which patients were able to sit alone, walk alone, and speak their first words (or the ages at which these developmental milestones were not yet reached) were obtained from caregivers or recorded at the time of clinical examination by A.G. and F.T. prior to initiating the rehabilitation program.

The age at “independent sitting” was defined based on caregivers’ reports; “independent walking” was defined as the age at which patients achieved the ability to walk without help or support for approximately four meters; and “first words” refers to the age at which the ability to say at least two intelligible words was recognized.

One additional male patient is discussed separately because of his distinctive genetic condition: ID259 showed a complex karyotype, and array-CGH demonstrated concurrent deletion and duplication events: 46,XY,del(5)(p15.33p15.31)(151,737–9,053,602×1) and dup(5)(p15.31p14.1)(9,181,474–27,188,057×3); the patient, therefore, carries three copies of the *TRIO* gene. His HC at birth (at the 38th gestational week) was 33 cm, and at age 5 years it was 51 cm; he was able to sit independently at 12 months, to walk at 24 months, and to say his first words at 18 months. The mdb also includes two pairs of twins carrying mosaic 5p deletions, who displayed discordant phenotypes even in the presence of a similar number of abnormal cells [16]; however, they were not included in this study and will be reported separately. 

In our cohort, the deletion size is 20.97 ± 8.87 Mb (Table 1), which is comparable to that reported by Nevado et al. [3], although we did not observe the significant differences between males and females they found (SI Table S8).

### 2.2. Overall Data Analysis

**Figure 1:**
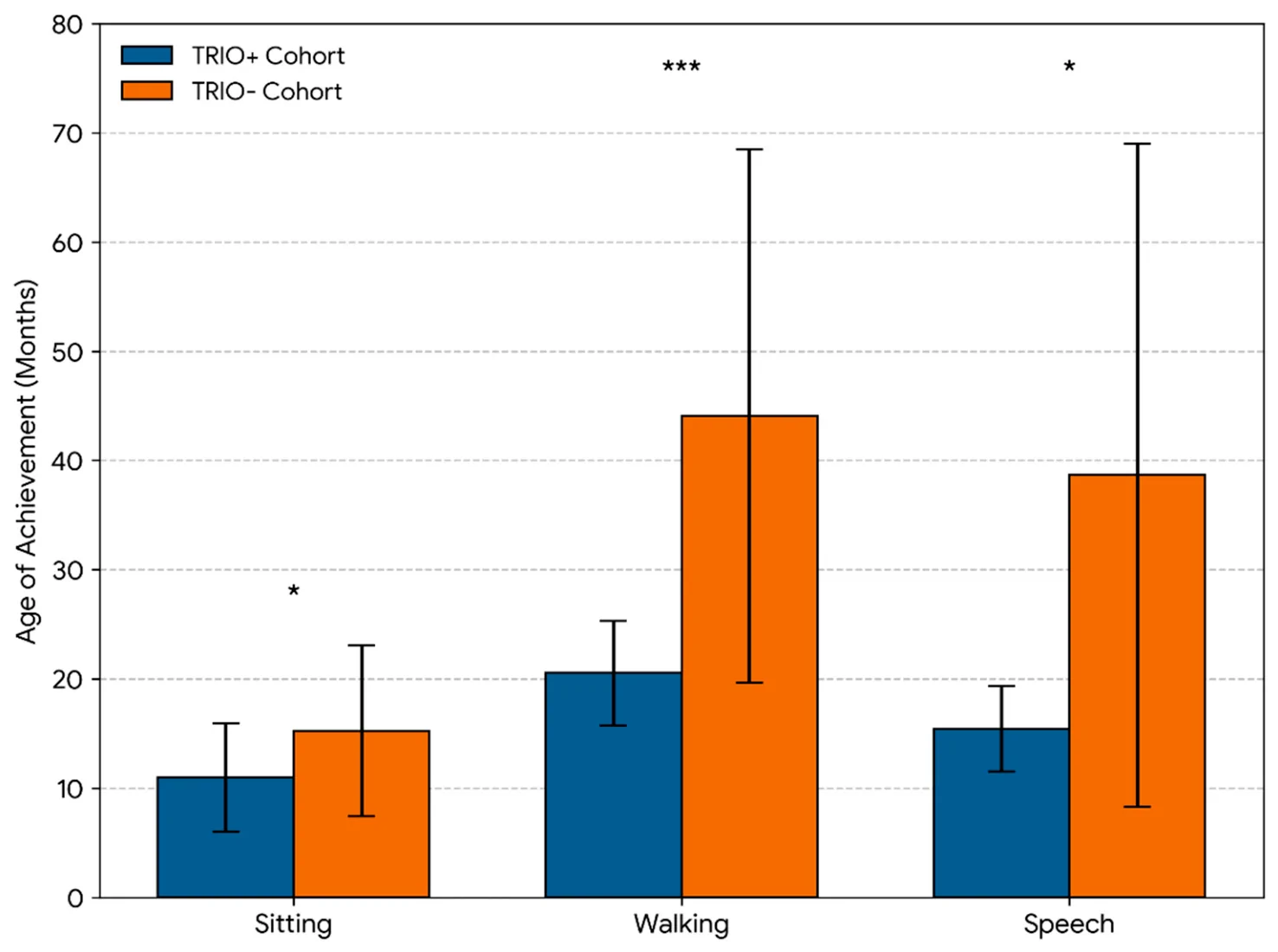
Neurodevelopmental Milestones and *TRIO* Gene Status: Comparison of Sitting, Walking, and Speech acquisition ages. *TRIO*+ patients demonstrate faster development in sitting, walking, and speech (see Table 2 for details and significance). *: *p* < 0.05; *** *p* < 0.001.

We first explored the role of *TRIO* on the clinical phenotype by comparing two cohorts, *TRIO*+/*TRIO*− vs. HC, sitting age, walking age, and speech separately (Table 2, Figure 1).

### 2.3. Multivariate Analysis

We examined the impact of *TRIO* (+/−), gestational age (weeks) and deletion size on HC and key developmental milestones. To evaluate the independent contribution of each factor, linear regressions were performed for each outcome variable against the three independent predictors (Table 3).

To determine whether *TRIO* status (+/−) or genetic quantity (deletion size) serves as a better predictor for phenotypic outcomes, a multiple linear regression analysis was conducted. This model pits both factors against one another across all major neurodevelopmental milestones and HC, and shows that the loss of the *TRIO* gene has a significant impact on gross motor delays.

Sitting: (*p* = 0.01); *TRIO* status is significantly more relevant than (*p* < 0.05) deletion size; loss of *TRIO* shifts sitting onset by +6.01 months.

**Figure 2:**
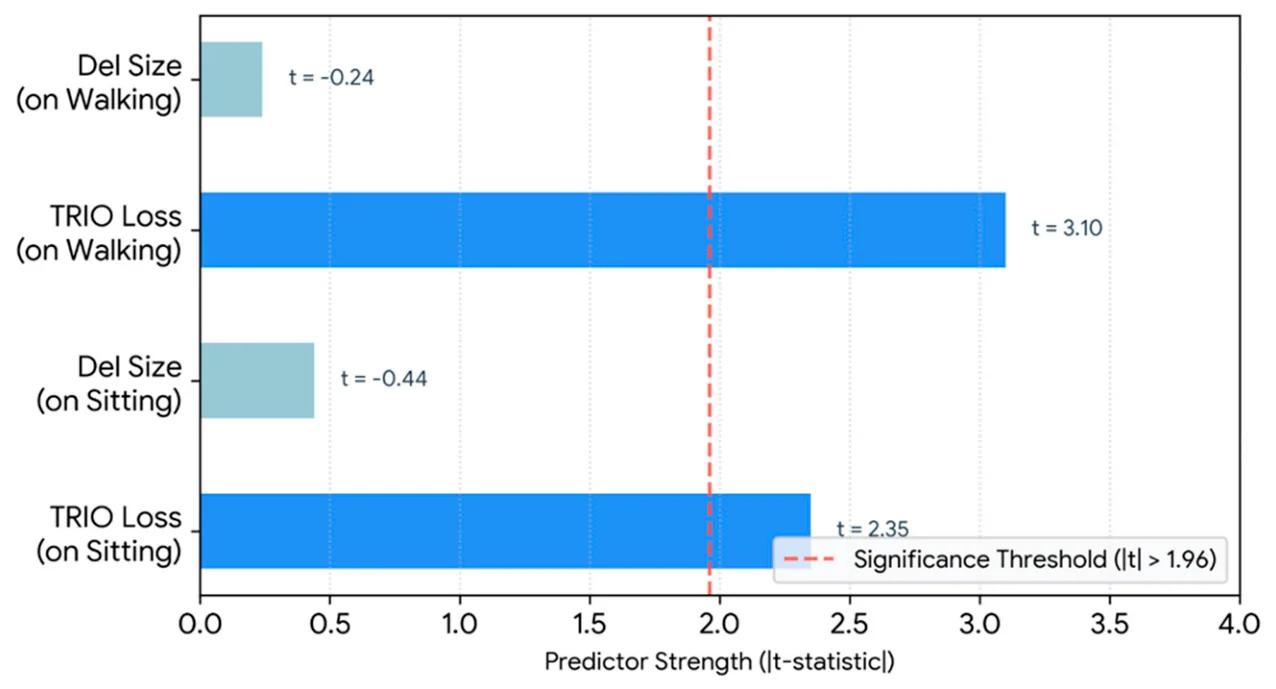
Standalone multi-model *t*-statistic layout evaluating the predictor strengths. Blue horizontal markers define the *TRIO* effect while faded blue markers plot the cumulative deletion size impact. The dashed red line indicates significance.

Walking: (*p* = 0.008); *TRIO* status is significantly more relevant than (*p* < 0.01) deletion size; loss of *TRIO* independently adds a delay of 23.97 months (~2 years) to independent walking (Figure 2).

Speech: beta coefficient for *TRIO* is high and positive, but the high SE dilutes the predictive effect of both features (SI Table S3).

Reduced HC at birth is independent of both *TRIO* content and deletion size, but associated with gestational age (Table 3). We then removed the “noise” of prematurity by performing a subgroup analysis restricted to full-term infants (37–41 weeks). Among them, infants with a *TRIO* (+) gene status display a mean HC that is 1.58 cm larger than their *TRIO* (–) counterparts (Table 4). In this subgroup, multivariate analysis hints that *TRIO* gene retention, when compared to deletion size and gestational age, significantly protects against reduced HC at birth (Table 4).

**Figure 3:**
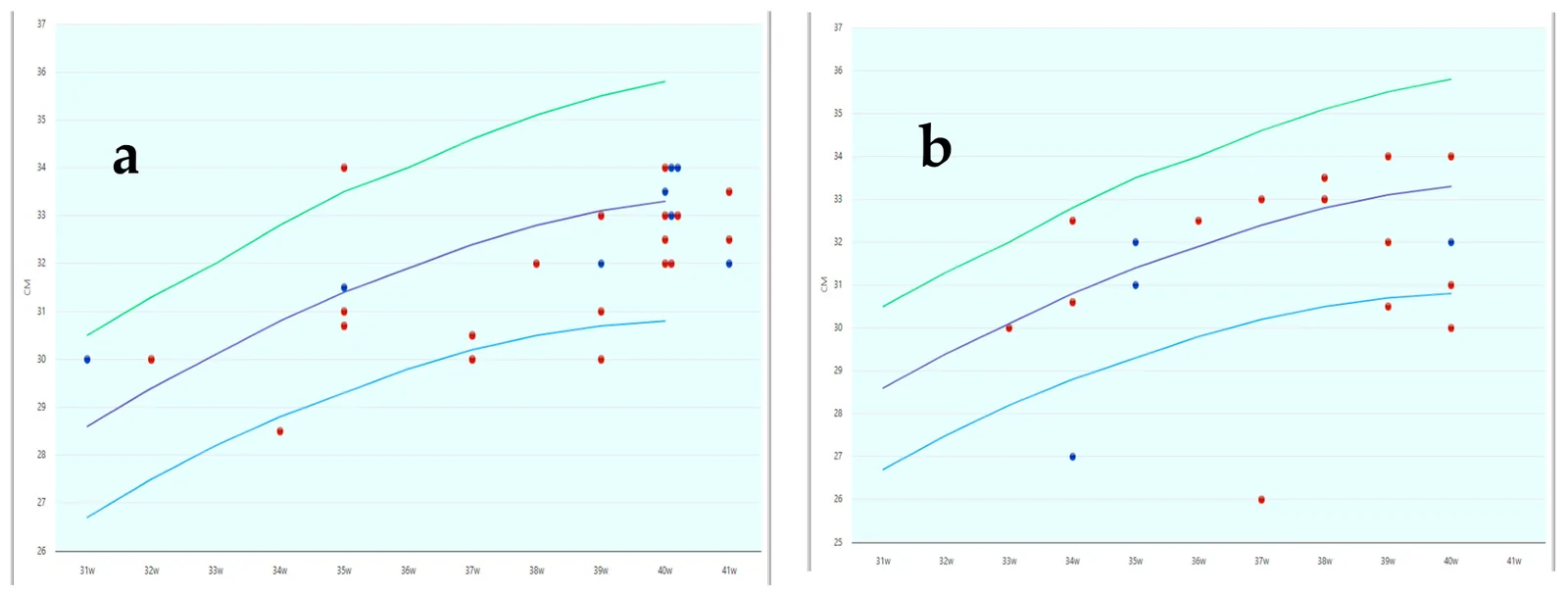
Distribution of HC at birth in 27 females (**a**) and 18 males (**b**) CdC patients; red circle, presence of one allele for the *TRIO* gene; blue circles, presence of two alleles for the *TRIO* gene; top line, middle line, bottom line, 98th, 50th and 2nd centile, respectively.

We completed the analysis of *TRIO* status on HC by plotting single measurements for each patient on growth charts centiles: in Figure 3, and Table 5 we show the distribution of HC measures available for 46 cases at various gestational ages at birth, alongside their position on different centiles (98th, 50th, and 2nd) as calculated in [13].

In females, the shortest gestational age in the cohort was observed in ID61 and ID20, who did not share any specific clinical features and carried respectively one and two *TRIO* alleles (SI Table S1 and Figure 3a); three cases with HC measurements below the 2nd centile (ID230, ID257 and ID260) carry typical 5p deletions (SI Table S1 and Figure 3a), and two of them show deletion sizes larger than the mean deletion size observed in both males and females with one *TRIO* allele. The third case, ID260, has a smaller deletion but paternal HC was at the lower end of the control range, whereas maternal HC was at the mean.

One case, ID108, had an HC above the 98th centile for CdC patients [14] and a complex karyotype: in addition to the 5p deletion, there was a deletion on chromosome 10 [del10(10q21.3: 68,290,259–68,443,894×1)]. The four genes included in the deletion (*PBLD*, *HNRNPH3*, *RUFY2*, *DNA2*) are not reported to influence HC. Parents of ID108 had HC measurements at the 72nd–75th centile for the normal population. 

Similarly, among males, ID2 and ID14 show HC measurements at very low centiles, but do not correlate with deletion size.

In Figure 3a, ID259 (who carries three copies of the *TRIO* gene) is not included: his HC (33 cm at the 38th week) (SI Table S1) is above the 50th percentile.

**Figure 4:**
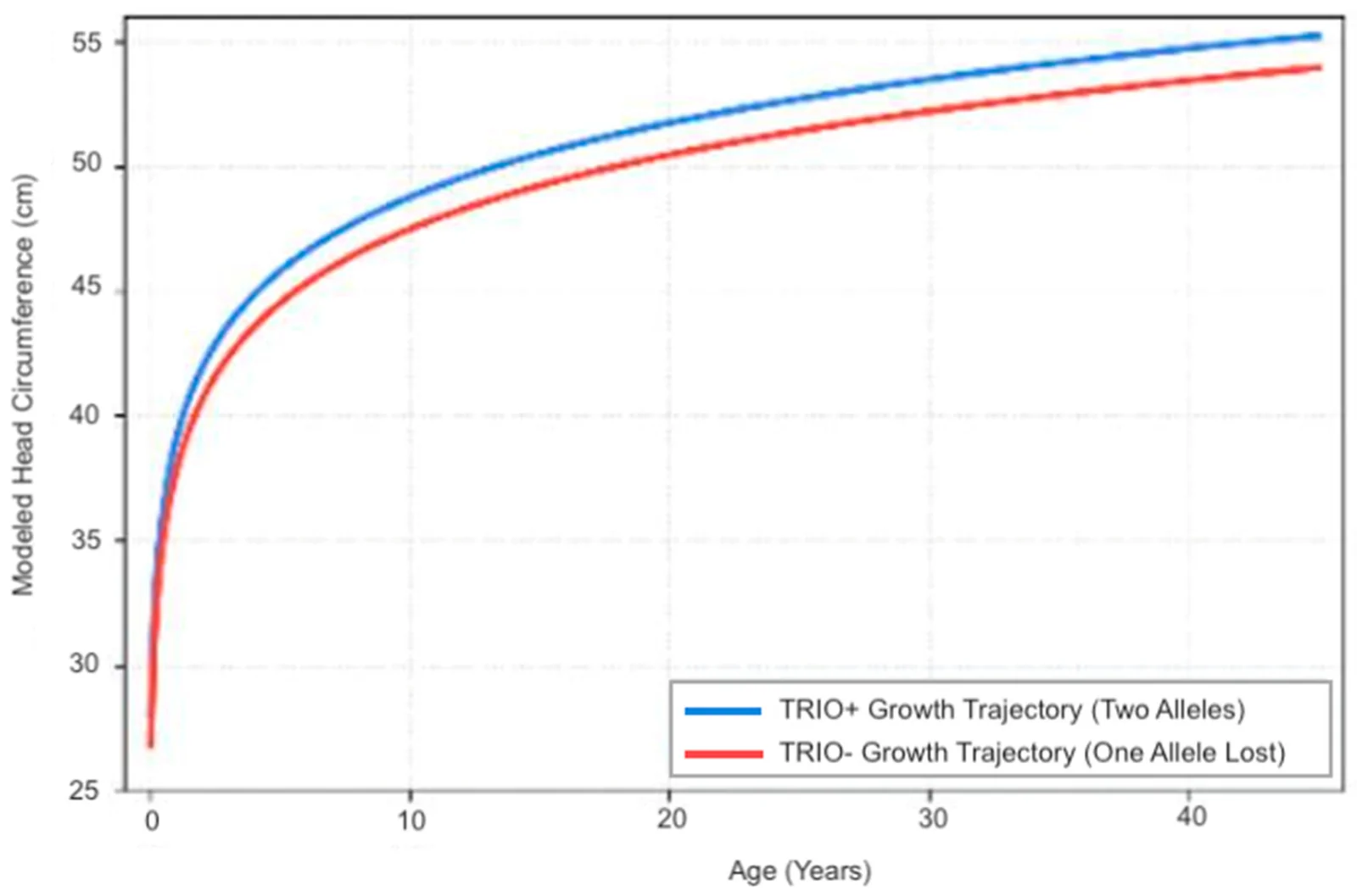
Extended longitudinal mixed-effects growth trajectories of head circumference (HC) in pediatric and adult populations stratified by *TRIO* status (n = 47). Non-linear growth trajectories modeled across a 45-year lifespan to encompass adult registry observations. The model utilizes 162 longitudinal timepoints from 47 unique CdCS patients (25 females, 87 measurements; 22 males, 75 measurements).

All available HC measurements were studied with a mixed-effects model and the results are entered in (Figure 4), The blue curve represents the estimated growth pathway for the *TRIO*+ cohort (two functional alleles), while the red curve tracks the *TRIO*− cohort (one allele lost), demonstrating consistently larger HCs and a slightly accelerated growth rate for TRIO+ group compared to the TRIO (−) group across all ages from birth to 40 years. This leads to a persistent difference in Hc in adult years.

Group Difference: subjects in the TRIO (+) group (whole group, males and females) tend to have systematically larger HC compared to the TRIO (−) group (*p* < 0.05 or *p* < 0.005).

Temporal Trend: while at birth the difference is minimal (~31.4 cm vs. ~32.1 cm), the gap increases with growth, and in adulthood, the average for the (+) group reaches 55 cm, compared to 52 cm for the (−) group.

Model Effectiveness: The use of random effects per patient allowed for correct handling of the variable number of observations, preventing subjects with more data from overly influencing the average (Figure 4, Table 6, and SI Table S2).

The mean deletion size in the overall cohort (n = 52) is entered in Table 1. The absolute deletion size does not differ between the sexes, as both the non-parametric Mann-Whitney U test (*p* = 0.730) and Welch’s (*t*)-test (*p* = 0.819) were non-significant. These results are in keeping with the hypothesis that clinical variations observed between males and females in phenotypic presentations cannot be attributed to a baseline difference in deletion size.

**Figure 5:**
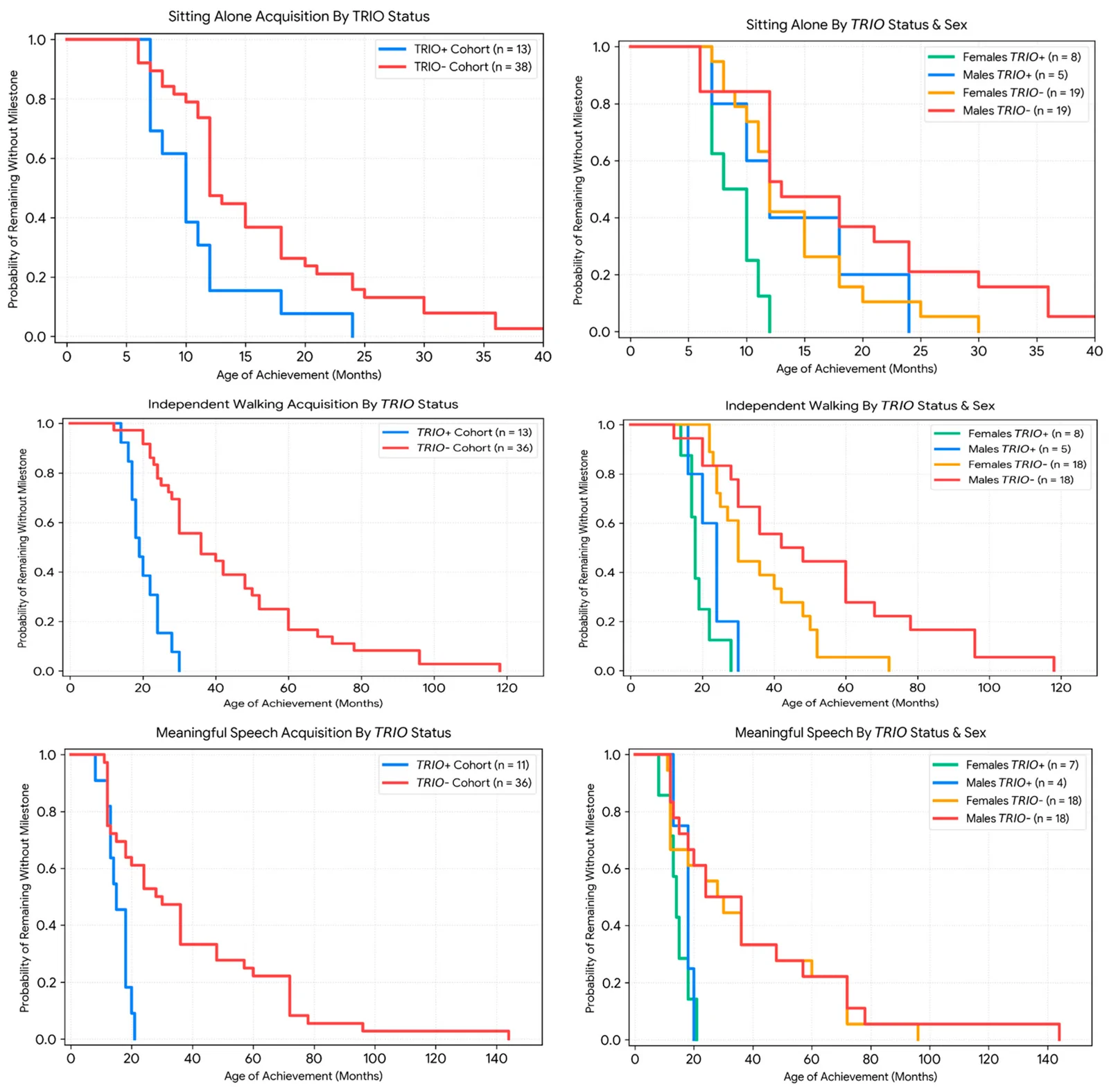
The curves reveal a rapid descent for the *TRIO*+ cohort (Left) for all the milestones considered, conversely, the *TRIO*− cohort exhibits a flattened curve, indicating delayed milestone acquisition.

For cases entered in SI Table S1, developmental milestones (age for independent sitting, walking, and first words) were analyzed using the Kaplan-Meier method to account for censored observations (milestones not yet reached): *TRIO* status significantly (Table 7) correlates with the age of achievement for these milestones. Specifically, the *TRIO* (−) group showed a trend toward delayed acquisition of motor and language skills compared to the *TRIO* (+) group (Figure 5, left column).

The sex stratified curves (Figure 5, right column) show that the lines representing both female *TRIO*+ and male *TRIO*+ decline almost synchronously, clustering tightly together in the lower-left sector of the graph. This indicates that the protective effect of having two *TRIO* alleles operates identically across sexes.

Similarly, (Female *TRIO*− and Male *TRIO*−) remain parallel as they stretch toward advanced developmental timelines. This pattern suggests a lack of sexual dimorphism in *TRIO* pathology. It confirms that gene loss delays motor milestones equally in both boys and girls.

Females reach the developmental milestones (sitting and walking) earlier than males in both the *TRIO* (+) and *TRIO* (−) groups (Figure 5), but the difference is not significant, while speech acquisition is superimposable in both sexes. Details in SI Table S5.

Case ID259 carrying 3 *TRIO* alleles achieved developmental milestones (sitting alone, walking independently, and speaking a few words) at ages 9 months, 26 months, and 14 months, respectively.

### 2.4. Complex Karyotypes

Among females with complex karyotypes carrying one *TRIO* allele, the mean ages for sitting and walking were very similar to those of the overall female group (SI Table S1), at 13.6 vs. 13.26 months and 38.66 vs. 36.38 months, respectively. In contrast, the age at first words was higher (72.0 vs. 42.88 months), mainly due to the delayed speech in ID213.

Among males with complex karyotypes carrying one *TRIO* allele, the mean ages for sitting and walking were also similar to those of the overall male group (SI Table S1), at 15.00 vs. 17.38 months and 60.00 vs. 52.44 months, respectively (note that only one case contributed to the walking age). The age at first words was slightly younger (30.00 vs. 40.33 months).

In males with complex karyotypes carrying two *TRIO* alleles, the mean ages for sitting and walking were comparable to those of the overall male group (17.00 vs. 14.23 months and 22.00 vs. 22.81 months, respectively), and the age at first words was similar (20.00 vs. 17.25 months).

The deletion size in cases with complex karyotypes did not show any peculiarities. As excluding these cases from the analyses of clinical data or deletion size did not alter the statistical results, they were retained in the overall cohort (details in SI Table S6).

The smallest HC measurement at birth, 26 cm (at 37 weeks), was observed in patient ID14, a *TRIO*− male; the patient’s deletion size (22.7 Mb) is comparable to other *TRIO*− males, whereas his developmental milestones were clearly delayed compared to the group means (SI Table S1). Several patients (IDs 17, 59, 85, 108, 234, and 274) shared a high HC (34 cm); however, their deletion sizes were highly heterogeneous (SI Table S1), ranging from 11.05 to 27.16 Mb, and did not correlate with HC measurements.

Regarding developmental milestones, the timing of early or late achievement was inconsistent within single cases. For instance, patients ID98 and ID100 exhibited an early age for sitting and first words, yet walking was delayed or not yet achieved. In contrast, patient ID42 demonstrated an early sitting and walking age, but delayed speech onset; notably, these patients all shared a large deletion size well above the group mean. Similarly, other patients with late developmental achievement presented with a wide range of deletion sizes, spanning from 11.83 to 33.13 Mb (SI Table S1). 

## 3. Discussion

The prevalence of CdCS among females is known to be higher than in males, ranging from 59% in the largest case series (n = 374) [14] to 66% in a more recent study (n = 70) [3]. However, no differences have been reported between ethnicities or across geographical areas, and no evidence supports any relevance of prenatal events or parental age. The reason for the higher female prevalence is still unclear.

In the mdb of A.B.C., 258 living patients are registered, with females (n = 142; 55%) being slightly more numerous than males, in agreement with the literature. Similarly, the subgroup selected for in the present study (28 females, 53.85%, and 24 males, 46.15%) shows the slightly higher prevalence of females observed in the mdb, which is coherent with unselected cohorts such as those in [3,14].

The overall comparison based on *TRIO* status (*TRIO*+ vs. *TRIO*−) showed significant differences for all developmental milestones (sitting, walking, and speech), demonstrating earlier achievement in *TRIO*+ cases, but the *TRIO* status difference was not significant for birth HC. This result was confirmed by multivariate analysis, in which *TRIO* (+) is the only variable reaching significance for sitting and walking, while in the case of speech, the independent effect is still present, but significance was not reached, likely due to high variance.

Our results suggest that, when considering the effect of a single gene, *TRIO*, its status, loss of one allele or retention of both alleles (SI Table S1), can be correlated to some developmental milestones and to HC in full-term babies.

This model, attempting to determine the effect of a single gene in cases with complex karyotypes with loss of hundreds of genes, may improve our ability to describe phenotypic changes in CdC patients, but can be applied to other chromosomal abnormalities too.

According to Nevado et al. [3], “a suspicion of putative cognitive and functional differences between male and female patients has been constantly suggested to us by parents”; moreover, the authors reported, “poorer functional scores and larger deletion sizes in females than in males using Ward’s cluster analysis.”

In our cohort (Figure 5), the ages at which independent sitting and walking were achieved were consistently more delayed in males than in females, regardless of whether patients carried one or two *TRIO* alleles; however, these differences were not statistically significant. Overall, in our sample of CdC patients, females did not exhibit poorer performance across the evaluated developmental milestones. While sex differences have been repeatedly observed in the literature, a clear explanation for this variance is still lacking. However, the reported evidence [3] and our own data (although not statistically significant) suggest that, in future studies of CdC patients, all developmental and functional parameters should be analyzed separately by sex. This approach will help verify if, in larger sample sizes or for different clinical outcomes, patient sex plays any significant role.

HC measurements were clearly distributed differently across the various centiles in both females and males, and correlated with the number of *TRIO* alleles (one or two) present in each patient: cases carrying a single *TRIO* allele consistently fall at lower centiles than those from cases carrying two *TRIO* alleles. Figure 3 and Figure 5 and SI Figure S2, suggesting the relevance of the number of *TRIO* alleles on the development of head size.

When a multivariate analysis was performed, it indicated that, at birth, gestational age is the strongest independent predictor of HC in the studied population (Table 3).

The relevance of *TRIO* gene status (+) or (−) as a genetic determinant clearly emerges when its effect is challenged in the full term subgroup 37–41 weeks (SI Table S4). The significant difference highlights that TRIO gene preservation mitigates microcephaly independently of total deletion size and minor variations in term gestational age. These results underscore the necessity of adjusting for gestational age when evaluating cranial growth deficits in genetic syndromes. When a mixed model is applied to the study of HC (Figure 4), again the *TRIO* (+) group consistently demonstrated larger HCs, confirming the relevance of the number of *TRIO* alleles on cranial growth. The fact that the curves do not diverge over time indicates that *TRIO* loss does not cause a progressive reduction of cranial growth with aging. The growth deficit is fully established during the early infantile brain-growth spurt, and once this baseline volumetric restriction occurs, the skull tracks a normal, log-transformed growth velocity (β = 4.31, *p* < 0.001) and remains permanently locked on a lower phenotypic track.

This observation is consistent with the genetics of the human pathologies associated with *TRIO* mutations [6]: pathogenic *TRIO* variants cluster in two hotspots that negatively (loss-of-function) or positively (gain-of-function) affect the activation of the downstream target RAC1, resulting in distinct developmental defects. Specifically, missense pathogenic variants in the spectrin repeat domain are associated with macrocephaly, whereas many truncating or missense variants in the GEFD1 domain are linked to microcephaly [6,7,8,9,17].

In turn, altered RAC1 activity levels correlate well with the head size of affected individuals, as Pengelly et al. demonstrated that pathogenic *TRIO* variants that result in reduced RAC1 activation are related to specific phenotypes, including microcephaly [8]. Furthermore, Reijnders and colleagues [18] reported a wide variation in HC associated with distinct (rare) pathogenic variants in the *RAC1* gene and showed that variants acting as dominant-negative alleles result in microcephaly.

Collectively, our findings support a role for *TRIO* in modulating the microcephaly characteristic of CdC, and the hypothesized mechanism is via dysregulated RAC1.

HC was reduced compared to normative curves in both males and females, at birth and in post-natal life (Figure 3 and SI Figure S2), with a slightly higher prevalence of reduction in females; however, the difference between sexes was not statistically significant, in contrast with previously reported data [3].

In our study, it appears that the loss of one *TRIO* allele mimics loss-of-function variants affecting the gene and correlates with a reduced HC.

However, no significant differences were found in the mean HC values between the various genotype groups, nor were significant correlations found between HC measurements and deletion sizes in any of the comparisons studied (SI Table S7).

These observations suggest that, when studying groups of patients, the loss of a large number of genes might be difficult to correlate with specific clinical features when using data expressed as mean ± SD values or standard correlation analyses. In contrast, careful observation of individual cases can reveal phenotypic effects, even considering the loss of single genes within the deletions.

In addition, when comparing mean HC between cases born at ≤37 weeks and those born at ≥38 weeks, we observe a significant difference, as expected. Notably, the level of significance was much higher in the group carrying a single *TRIO* allele, consistent with the hypothesis that carrying two *TRIO* alleles is associated with a larger HC (and thus smaller differences), potentially augmenting the effect of gestational age alone.

When analyzed separately by sex, the effect of the number of *TRIO* alleles on HC is more pronounced in females (Figure 3a,b and SI Figure S2a,b) than in males. Specifically, 62% of females and 50% of males carrying two *TRIO* alleles were above the 50th centile for CdC-specific curves [14]. Significant differences were observed only in females. The lack of a significant difference in males may reflect the smaller sample size, although the influence of sex cannot be ruled out completely [3].

### TRIO (+/−), Deletion Size and Developmental Delay

The impacts of *TRIO* (+/−), gestational age (weeks), and deletion size on key developmental milestones were analyzed by multiple linear regression for Sitting, Walking, and Speech. The results align with the hypothesis that a functional *TRIO* (+) genotype could be relevant for earlier achievements of the motor milestones considered, while neither HC nor gestational age impacts their timing.

Multivariate analysis suggests that the number of alleles for the *TRIO* gene is a relevant and independent predictor of neurodevelopmental motor outcomes (Figure 2 and SI Table S3). When speech is considered, the beta coefficient for *TRIO* is still high and positive, but the high SE dilutes the predictive effect (SI Table S3). These results suggest that clinical prognosis should prioritize the mapping of specific critical genes, such as *TRIO*, over simple quantification of lost genetic material.

Interestingly, once *TRIO* status is accounted for, birth HC and gestational age do not significantly predict the timing of milestones in this cohort, in keeping with the hypothesis of the relevance of *TRIO* deletion on neurodevelopmental phenotype.

We did not find a correlation between the deletion size and an early or late age of onset of developmental milestones (SI Table S9). In a previous study [19], the relationship between deletion size and phenotypic severity in CdC was reviewed, noting that earlier studies produced conflicting results [20,21]. Other authors suggested that focusing on the identification of “critical regions” might be a more informative approach [22,23], and that only a weak relationship between developmental delay and deletion size can be found [24].

More recently, Villa et al. [25] identified a smaller region of overlap (SRO) for pontine hypoplasia in a cohort of 14 patients, even when this SRO was part of deletions of very different sizes.

Our data confirm that, when considering very specific developmental milestones—such as age at autonomous sitting, walking, and first words—the impact of the allelic loss of specific genes should be taken into account, a possibility that was previously underestimated.

The relevance of single-gene loss in the context of deletions is well exemplified, for instance, by 7p11.2 Microdeletion and the *RAI1* Gene. Smith-Magenis Syndrome (SMS) is typically caused by a 3.5 Mb interstitial microdeletion on chromosome 17p11.2. While this common microdeletion encompasses multiple genes, molecular dissection and the discovery of patients with de novo point mutations have definitively proven that the haploinsufficiency of a single gene, Retinoic Acid Induced 1 (*RAI1*), is the primary driver responsible for the core clinical phenotype [26].

Indeed, as shown in (SI Table S9), differences in deletion size are highly statistically significant, as expected, when comparing FT+/FT– or MT+/MT– groups. However, a highly significant difference in deletion size is not always accompanied by a corresponding difference in the age of attainment of developmental milestones. One possible interpretation is that deletion size alone, not accounting for the loss of specific genes, may be insufficient to explain particular clinical features.

A significant example supporting this notion comes from a recent study on rats: the authors defined a region whose deletion causes a “5p-like” phenotype and demonstrated that AAV-mediated gene replacement of *Ctnnd2* alleviates cognitive impairment in CdCS rats [27]. Importantly, their work illustrates how the pathological effect of deleting individual genes on specific biological functions can be identified, as well as how these deficits may be ameliorated by targeted gene replacement.

Regarding neurological development and the effect of single genes, the most common 5p deletion involves two genes expected to play critical roles in human brain development, namely *CTNND2* and *SEMA5A* [28,29,30]. Based on known and predicted functions of these genes, it has been proposed that their heterozygosity may explain the neurological and cognitive deficits in CdC patients.

*CTNND2* encodes the junction protein δ-catenin, which is expressed early in neuronal development and involved in cellular motility [31,32]. The role of *CTNND2* in the CdCS phenotype is supported by clinical observation of patients [33,34], studies in animal models [27,28,29,30], and its role as an upstream regulator of Rho-GTPases [32,35], which are key controllers of the dynamics of the neuronal cytoskeleton.

*SEMA5A* encodes a protein implicated in axonal pathfinding and neurogenesis, which is mainly expressed in the hippocampus [36,37]. Studies in animal models have shown the importance of *SEMA5A* for cortical development through the fine control of radial migration [38]. Functional studies on human brain organoids will unravel the exact role of these two genes for the delayed neurological and cognitive development of CdC children.

It should also be noted that definitions such as “developmental delay” and qualifiers like “mild” or “severe”, while straightforward in clinical usage, are inherently imprecise. In addition, the impact of pathogenic variants in genes located outside of the 5p region, as well as epigenetic changes, are often not considered.

Holland et al. [39] showed that, beyond the primary effect of 5p haploinsufficiency, Cri du chat is associated with a genome-wide DNA-methylation signature: differentially methylated CpGs were enriched in neurodevelopmentally relevant pathways and in genomic regions related to key clinical features (e.g., developmental delay, microcephaly); these enrichments remained detecTable even after excluding CpGs on chromosome 5 (including 5p), thus supporting a trans-acting regulatory/epigenomic component rather than a purely local, cis effect. Consistently, widespread methylation alterations across the genome in a toddler with 5p deletion were reported [40], with a noTable over-representation of changes in regulatory regions, suggesting that individual-level differences in downstream epigenetic remodeling may act as phenotypic modifiers and help explain why patients with broadly similar 5p deletions can still diverge clinically.

## 4. Materials and Methods

The A.B.C. mdb comprises 258 living patients, with a mean age of 28 years and 8 months (range: 2 years and 7 months to 61 years and 4 months). Among them, 116 are males (44.9%; mean age 29 years and 7 months, range: 2 years and 7 months to 59 years and 3 months) and 142 are females (55.1%; mean age 27 years and 11 months, range: 3 years and 2 months to 61 years and 4 months).

Cytogenetic and array-CGH analyses were performed in different Italian centers, all of them with outstanding experience in the field, and were repeated and updated when indicated. Deletion size was referred to hg19 for all cases, but [ID20; ID23; ID45; ID48; ID56] referred to hg18, and [ID29; ID75; ID88; ID89] referred to hg17. To ensure consistency across all samples, legacy genomic coordinates mapped to older human genome assemblies—specifically hg17 (NCBI Build 35) and hg18 (NCBI Build 36)—were systematically converted to the hg19 (GRCh37) reference assembly. The liftover procedure was executed utilizing the UCSC LiftOver tool (University of California, Santa Cruz; ucsc.edu). Standard conversion parameters were maintained, requiring a minimum ratio of bases that must remap equal to 0.95. Following coordinate remapping, the exact genomic size of each microdeletion was recalculated by subtracting the updated proximal breakpoint coordinate from the distal breakpoint coordinate; conversion did not change the definition of *TRIO* + or *TRIO*−; in Table 1, all data are as for hg19.

Descriptive statistics (mean, standard deviation, minimum, and maximum values) were calculated for birth HC and psychomotor milestone achievement ages (sitting alone, walking alone, and speech) using the website “Social Science Statistics” (https://www.socscistatistics.com/tests/chisquare2/default2.aspx, accessed on 15 February 2026). Due to the non-Gaussian distribution and presence of phenotypic outliers inherent to developmental metrics, continuous variables were compared between the *TRIO*+ and *TRIO*− cohorts using the non-parametric Mann-Whitney U test (also known as the Wilcoxon rank-sum test) [41]. Pairwise deletion was applied to handle missing or unacquired clinical entries (--, n.ac., or n.av.) to maximize the usage of available data.

To rigorously model the developmental velocity and cumulative probability of developmental milestone achievement (sitting alone, independent walking, and speech) across chronological age, a time-to-event survival analysis framework was implemented utilizing the non-parametric Kaplan-Meier estimator [42].

For each sub-model, the operational timeline began at birth (month 0), and chronological age at milestone achievement (expressed in months) was designated as the target “event.” To eliminate selection bias from missing follow-up entries or patients who had not yet successfully completed the milestone, and to optimize statistical power, these instances (documented as --, n.av., or n.ac. in the raw database) were rigorously integrated as right-censored observations. Censored individuals contributed mathematically to the total pool of patients “at risk” of non-achievement up to their maximum known longitudinal tracking threshold, without introducing false event entries. Statistical divergence between the resulting survival steps was mathematically verified using the Log-Rank (Mantel-Cox) test, which evaluates the global equivalence of achievement curves under a chi-square distribution.

To evaluate the longitudinal trajectory of HC expansion across the extended lifespan (up to 45 years of age) while controlling for repeated measures within the same individual, a Linear Mixed-Effects Model (LMM) was implemented. Because human cranial growth follows an exponentially decaying velocity—characterized by rapid infant expansion followed by a protracted plateau—chronological age was modeled using a log-transformation: log({Age in Years} + {month}). This approach ensures biological alignment with non-linear cranial development and stabilizes residual distribution.

To isolate the independent contribution of specific gene content (TRIO) from global structural mutation volume and baseline physiological development, total deletion size (standardized in Megabases, Mb) and gestational age at birth (weeks) were integrated into the fixed-effects matrix as critical structural covariates.

To mathematically account for the clustered dependency of longitudinal repeated measures and the unbalanced number of observations per patient, a random-intercept framework was utilized. Individual patients were entered as random effects, allowing each subject to possess a unique, baseline deviation from the global population intercept. Fixed-effects coefficients (beta), standard errors (SE), and corresponding z-statistics were calculated using maximum likelihood estimation. Missing data clusters were handled via pairwise deletion to maximize data density across the 141 individual longitudinal tracking timepoints.

## 5. Conclusions

We sought to determine whether a genotype–phenotype correlation could be observed in a cohort of CdC patients when considering a single 5p gene present in one or two copies. The selected gene, *TRIO*, is known to cause microcephaly and a complex pattern of developmental delay when mutated.

Our results suggest that such a correlation may be detectable. While all CdC patients exhibit reduced head size, we observed that HC tended to be smaller in patients carrying only one *TRIO* allele compared to those with two alleles.

Moreover, patients with only one *TRIO* allele generally showed greater delays in the developmental milestones considered, specifically, autonomous sitting, walking, and age at first words.

For these developmental outcomes, we also observed small differences between males and females, highlighting the importance of considering sex as a variable in both clinical description and rehabilitation planning in future studies.

At the functional level, the available evidence and our new results suggest that haploinsufficiency of *TRIO* may be a relevant genetic factor of more pronounced microcephaly in a subset of CdC patients. This observation underscores the value of further functional studies on the role of *TRIO* in human brain development and neural progenitor cell proliferation.

A gene-dosage approach to study GPCs has been extensively studied in microdeletions, as in [26]; our work confirms its relevance and suggests that it should become a routine method for future research and clinical practice.

### Limitations

Limitations of the study include the reliance on some data obtained from caregivers and variations in the ages at which the clinical data were observed. While these constraints inherently stem from analyzing data collected within the database of a charity organization such as A.B.C., this study (like many others investigating rare diseases) was made possible on such a large cohort only by exploiting the vast amount of information collected by the family association. A further limitation derives from the imbalance between the *TRIO*+ (n = 12) and *TRIO*− (n = 40), which might introduce some bias in statistical calculations, again strictly linked to the availability of data. In future studies, certainly, functional studies as well as comparison with other genes present in the deletion will be considered.

## Figures and Tables

**Table 1:** Summary of 5p genomic deletion sizes.

Cohort Group	Cases (n)	Mean Deletion Size (Mb)	(SD)
Whole Group	52	20.97	8.87
Males	24	21.21	7.91
Females	28	20.77	9.74

Lengths of the chromosome 5p loss are expressed in Megabases (Mb) across the entire target cohort and stratified by biological sex (24 males, 28 females). SD: Standard deviation.

**Table 2:** Comparison of Clinical and Developmental Parameters Based on *TRIO* Gene Status.

Clinical Parameter	*TRIO*+ Mean ± SD	*TRIO*− Mean ± SD	Student’s *t* Test (*p* Values)	Mann–Whitney U Test (*p* Values)
Head Circumference (cm)	31.83 ± 1.93 (n = 12)	31.67 ± 1.77 (n = 34)	0.801 ^	0.696 ^
Sitting Onset (months)	11.00 ± 4.97 (n = 13)	15.27 ± 7.83 (n = 37)	0.030 *	0.033 *
Walking Onset (months)	20.53 ± 4.79 (n = 13)	44.08 ± 24.41 (n = 36)	<0.0001 ***	0.00003 ***
Speech Onset (months)	15.45 ± 3.91 (n = 11)	38.66 ± 30.33 (n = 36)	0.0001 ***	0.023 *

*TRIO*+: two alleles; *TRIO*−: one allele (+); n: number of cases; ^: not significant; *: *p* < 0.05; *** *p* < 0.001.

**Table 3:** Results of linear regression.

Dependent Variable (Outcome)	Predictor Variable	Coefficient β	SE	*p*-Value	Significance
(HC)	TRIO (+) status Gestational Age (weeks) Deletion Size (Mb)	+0.62 +0.19 −0.01	0.61 0.08 0.03	0.316 0.024 0.697	n.s. *p* < 0.05 n.s.
Sitting Onset (Months)	TRIO (+) status Gestational Age (weeks) Deletion Size (Mb)	−14.73 −0.58 +0.33	5.37 0.70 0.25	0.010 0.413 0.195	*p* < 0.05 n.s. n.s.
Walking Onset (Months)	TRIO (+) status Gestational Age (weeks) Deletion Size (Mb)	−24.81 −0.89 +0.47	8.85 1.15 0.41	0.008 0.443 0.259	*p* < 0.01 n.s. n.s.
Speech Onset (Months)	TRIO (+) status Gestational Age (weeks) Deletion Size (Mb)	−21.36 −1.41 +0.42	11.45 1.50 0.54	0.071 0.354 0.439	n.s. n.s. n.s.

HC: Head circumference; Mb: megabase; n.s.: non-significant.

**Table 4:** Multivariate Regression Model for HC in Full-Term Subgroup (37–41 Weeks).

Variable	Coefficient (β)	SE	*p*-Value
TRIO Status (+ vs. −)	+1.58 cm	0.68	0.027 *
Deletion Size (Mb)	−0.016 cm	0.011	0.158 ^
Gestational Age (Weeks)	+0.14 cm	0.18	0.442 ^

SE: Standard Error; ^: non-significant; *: *p* < 0.05.

**Table 5:** Distribution of *TRIO* (+) and (−) CdC patients in growth charts.

	*TRIO*+	n/Total (+) > 50th Centile	*TRIO*−	n/Total (−) < 50th Centile
Females (n = 27)	8 (29.6%)	5/8 (62.5%)	19 (70.3%)	15/19 (78.9%)
Males (n = 18)	4 (22.2%)	1/4 (25%)	14 (77.7%)	7/14 (50%)

Distribution of *TRIO* (+) and (−) females and males above or below the 50th centile.

**Table 6:** Results of mixed effect model for *TRIO* status on HC.

	Difference (cm)	Significance
*TRIO* (+) vs. *TRIO* (−):	+2.55	*p* < 0.005
Males vs. Females	+0.81	n.s.
Males: *TRIO* (+) vs. *TRIO* (−)	+2.17	*p* < 0.05
Females: *TRIO* (+) vs. *TRIO* (−)	+2.55	*p* < 0.005

Differences in cm for the various *TRIO* (+) and (−) groups; n.s.: non sgnificant.

**Table 7:** Log-Rank (Mantel-Cox) mathematical parameters across developmental milestones.

Developmental Milestone	Statistical Stratification Axis	Chi-Square	(df)	*p*-Value	Significance
Sitting Alone	TRIO+ vs. TRIO− Females: *TRIO*+ vs. *TRIO*− Males: *TRIO*+ vs. *TRIO*− Interaction: Sex Effect	7.91 4.12 3.84 0.21	1 1 1 1	0.0049 0.0424 0.0500 0.6468	*p* < 0.01 *p* < 0.05 *p* = 0.05 n.s.
Independent Walking	TRIO+ vs. TRIO− Females: *TRIO*+ vs. *TRIO*− Males: *TRIO*+ vs. *TRIO*− Interaction: Sex Effect	12.18 6.54 5.72 0.08	1 1 1 1	0.0005 0.0105 0.0168 0.7773	*p* < 0.001 *p* < 0.05 *p* < 0.05 n.s.
Speech/First Words	TRIO+ vs. TRIO− Females: *TRIO*+ vs. *TRIO*− Males: *TRIO*+ vs. *TRIO*− Interaction: Sex Effect	4.82 2.64 2.21 0.15	1 1 1 1	0.0281 0.1042 0.1371 0.6985	*p* < 0.05 n.s. n.s. n.s.

Chronological time-to-event comparisons evaluating the cumulative achievement speeds of motor (sitting and independent walking) and cognitive-linguistic (speech) boundaries. Metrics track the global genetic impact of TRIO gene dosage (12 *TRIO*+ vs. 40 *TRIO*−) alongside sex-stratified subsets (28 females, 24 males). n.s.: non significant.

## Data Availability

The original contributions presented in this study are included in the article/Supplementary Materials. Further inquiries can be directed to the corresponding authors.

## Supplementary Materials

The following supporting information can be downloaded at: https://www.mdpi.com/article/10.3390/ijms27219400/s1.

## Abbreviations

The following abbreviations are used in this manuscript:

HC	Head Circumference
mdb	Main database
A.B.C.	Italian charity Associazione Bambini Cri du Chat
CdCS	Cri du Chat Syndrome
GPCs	genotype-phenotype correlations
