# Supplementary material for: Genotype-Phenotype Correlation in Cri du Chat Patients Carrying One or Two Copies of the TRIO Gene
Source: Int J Mol Sci. 2026 Oct 22;27(21):9400. doi: 10.3390/ijms27219400 (PMC13763483; doi:10.3390/ijms27219400)
Supplement: Supplementary file 1 [file ijms-27-09400-s001.zip › ijms-4289541-supplementary.pdf]

Supplementary Information (SI)

SI Figure S1

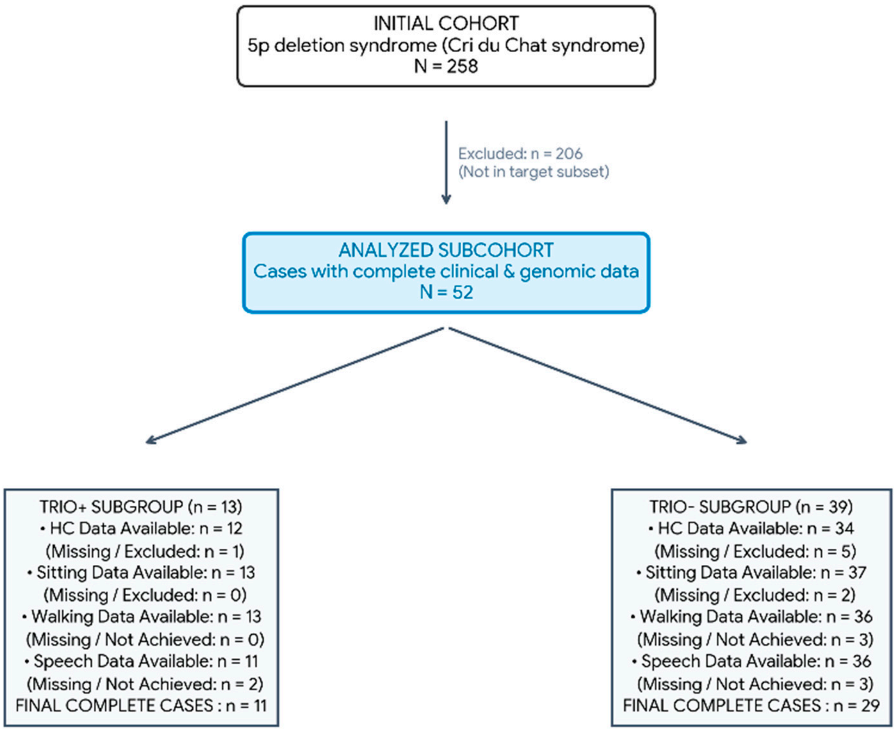

Figure S1. Compact flow chart of patient selection. Breakdown of the cohort reduction from the initial screening (N = 258) to the target subcohort (N = 52). Developmental milestones availability (*sitting, walking, and speech*) and sequential exclusions are detailed inside each respective genomic *TRIO* subgroup box.

SI Table S1

Table S1. List of cases included in the study. *: case with complex karyotype; ID-patient: patient identification number in the main database; Sex: F = female, M = male; *TRIO*: "-" = one allele (deleted); "+" = two alleles; Weeks: gestational age at delivery (weeks); HC: head circumference at birth; distalbp: distal breakpoint; proxbp: proximal breakpoint; del: size of the deleted region; sitting: age at autonomous sitting; walking: age at autonomous walking; speech: age at acquisition of at least two intelligible words; n.ac.: not acquired; "—": data not available.

case	ID	sex	TRIO	weeks	hc	distalbp	proxbp	del	sitting	walking	speech
1	163	F	-	37	30.50	151737	25674751	25523014	9	25	12
2	20	F	+	31	30.00	148243	11948243	11800000	12	22	12
3	21	M	-	40	31.00	151737	28163810	28012073	12	42	18
4	235	F	+	41	32.00	151737	7418199	7266462	10	18	14
5	224	M	-	39	31.00	26142	23266064	23239922	24	36	36
6	29	F	-	39	31.00	8697566	21107608	12410042	18	22	72
7*	33	F	-	40	33.00	151737	18909607	18757870	12	50	12
8*	100	M	-	n.av.	n.av.	151737	31810133	31658396	6	n.ac.	12
9	39	F	-	41	32.50	151737	17812007	17660270	7	30	12
10	86	M	-	34	32.50	1498180	19955760	18497580	n.av.	48	36
11	42	M	-	33	30.00	22179	32437680	32415501	6	12	24
12	254	M	-	38	33.50	151737	26287349	26135612	15	28	13
13	230	F	-	39	30.00	151737	28946166	28794429	12	72	36
14	257	F	-	34	28.50	151737	33287601	33135864	15	n.ac.	72
15	258	F	+	40	33.00	22149	11162737	11140588	7	17	21
16	56	F	-	41	33.50	75148	17766543	17691395	18	52	18
17	88	F	-	40	32.00	1651737	16585936	14934199	11	30	30
18	12	F	-	40	33.00	151737	17924904	17773167	30	52	60
19	48	M	-	n.av.	n.av.	148243	21305214	21156971	30	60	15
20	260	F	-	37	30.00	13307	16192603	16179296	8	22	28
21	59	F	-	40	34.00	38139	17509888	17471749	12	24	48
22*	2	M	+	34	27.00	151737	8124532	7972795	10	20	20
23*	110	M	-	37	33.00	151737	22246071	22094334	24	60	48
24	14	M	-	37	26.00	151737	22853652	22701915	36	60	72
25	50	M	-	34	30.60	151737	17924904	17773167	36	96	24

26	16	M	-	38	33.00	151737	29607531	29455794	12	118	144
27	17	M	-	39	34.00	6910791	34073756	27162965	12	20	12
28	76	M	-	n.av.	n.av.	151737	24001674	23849937	18	78	78
29	87	F	-	39	33.00	151737	25881161	25729424	11	36	96
30	239	F	-	40	32.50	151737	29152795	29001058	8	30	12
31	61	F	-	32	30.00	151737	14215420	14063683	15	27	24
32	45	M	+	40	32.00	75148	12691760	12616172	12	24	18
33	274	F	+	40	34.00	55550	11113718	11058168	11	19	13
34	75	M	-	40	30.00	1651737	34938070	33286333	18	68	n.ac.
35	98	M	-	39	30.50	151737	25674751	25523014	6	36	12
36	214	M	+	35	32.00	22179	13250086	13027907	18	30	18
37	280	F	+	39	32.00	55550	5046120	4990570	8	28	18
38*	213	F	-	n.av.	n.av.	151737	26594000	26542263	20	42	11
39*	95	M	+	n.av	n.av.	151737	11985200	11833463	24	24	n.ac.
40	137	F	-	38	32.00	151737	28946166	28788429	--	--	--
41	215	M	-	36	32.50	151737	32679377	32527640	21	96	72
42	51	F	+	40	33.50	151737	8124532	7972795	10	18	--
43	34	F	-	40	32.00	151737	31535885	31384148	10	40	36
44	46	M	-	39	32.00	9255340	19362945	10107605	12	20	20
45	85	M	-	40	34.00	9843524	24976470	15132946	13	30	36
46*	108	F	-	35	34.00	151737	25674751	26523014	9	24	72
47	89	F	-	35	30.70	4028280	18864601	14656321	15	23	n.ac.
48	232	M	+	35	31.00	22178	5539182	5517004	7	16	13
49	233	F	+	35	31.50	22178	5539182	5517004	7	17	15
50	234	F	+	40	34.00	22178	5539182	5517004	7	14	8
51	23	F	-	35	31.00	148243	26103882	25955639	12	48	12
52	1	M	-	36	n.av.	488960	15287349	14798390	12	30	57

SI Table S2

Table S2. Longitudinal Head Circumference (HC) Measures; Id-patient: patient identification number in the "main database"; sex: F > female; M > male; *TRIO*: "-": one allele; "+": two alleles; hc: head circumference; age: m: months; y: years; 16 > 27y : records taken between ages 16 and 27 (y,a>); 28 > 39y: records taken between ages 28 and 39 (y,b); >=40y: records taken at or after age 40 (y,c).

<table>
<tr><th>ID-Patient</th><th>Sex</th><th>TRIO</th><th>Age (m: months; y: years) & Corresponding HC (cm)</th></tr>
<tr><td>163</td><td>F</td><td>-</td><td>0m: 30.50 | 4y+6m: 43.50</td></tr>
<tr><td>20</td><td>F</td><td>+</td><td>0m: 30.00 | 7y: 49.00 | 11y: 50.00 | 16y-27y: 52.00</td></tr>
<tr><td>21</td><td>M</td><td>-</td><td>0m: 31.00 | 14y: 47.30 | >=40y: 49.50</td></tr>
<tr><td>235</td><td>F</td><td>+</td><td>0m: 32.00 | 11y+6m: 51.40 | 16y-27y: 53.00</td></tr>
<tr><td>224</td><td>M</td><td>-</td><td>4y: 44.50 | 8y+6m: 46.00 | 10y: 46.50</td></tr>
<tr><td>29</td><td>F</td><td>-</td><td>0m: 31.00 | 6y: 45.50 | 8y: 47.00</td></tr>
<tr><td>33</td><td>F</td><td>-</td><td>0m: 33.00</td></tr>
<tr><td>100</td><td>M</td><td>-</td><td>12m: 45.20 | 24m: 46.50 | 16y-27y: 51.50</td></tr>
<tr><td>39</td><td>F</td><td>-</td><td>0m: 32.50 | 3y: 44.00</td></tr>
<tr><td>86</td><td>M</td><td>-</td><td>0m: 32.50 | 7y: 49.30 | 28y-39y: 54.50</td></tr>
<tr><td>42</td><td>M</td><td>-</td><td>0m: 30.00 | 7y+6m: 47.00 | 14y: 51.00</td></tr>
<tr><td>254</td><td>M</td><td>-</td><td>0m: 33.50 | 3m: 38.00 | 24m: 44.30</td></tr>
<tr><td>230</td><td>F</td><td>-</td><td>0m: 30.00 | 3m: 33.50 | 6y: 44.00</td></tr>
<tr><td>257</td><td>F</td><td>-</td><td>0m: 28.50 | 7m: 36.50 | 6y: 45.00</td></tr>
<tr><td>258</td><td>F</td><td>+</td><td>0m: 33.00 | 9m: 43.50 | 5y: 48.50</td></tr>
<tr><td>56</td><td>F</td><td>-</td><td>0m: 33.50 | 24m: 42.50 | 8y: 48.00 | 16y-27y: 49.00</td></tr>
<tr><td>88</td><td>F</td><td>-</td><td>0m: 32.00 | 16y-27y: 49.00 | >=40y: 49.00</td></tr>
<tr><td>12</td><td>F</td><td>-</td><td>0m: 33.00 | 6y+6m: 47.00 | 10y: 48.00 | >=40y: 50.50</td></tr>
<tr><td>48</td><td>M</td><td>-</td><td>3m: 38.50 | 4y: 47.00</td></tr>
<tr><td>260</td><td>F</td><td>-</td><td>0m: 30.00 | 6m: 34.00 | 24m: 43.00</td></tr>
<tr><td>59</td><td>F</td><td>-</td><td>0m: 34.00 | 4m: 38.00 | 18m: 44.00 | 15y: 52.00 | 16y-27y: 55.00</td></tr>
<tr><td>2</td><td>M</td><td>+</td><td>0m: 27.00 | 3m: 32.50 | 6m: 38.00 | 4y: 44.00 | 10y: 47.00 | 12y: 47.70</td></tr>
<tr><td>110</td><td>M</td><td>-</td><td>0m: 33.00 | 8m: 41.50 | 4y+6m: 46.50 | 7y+6m: 47.50 | 10y: 48.50</td></tr>
<tr><td>14</td><td>M</td><td>-</td><td>0m: 26.00 | 5y: 44.50 | 15y: 49.00 | 28y-39y: 51.00</td></tr>
<tr><td>50</td><td>M</td><td>-</td><td>0m: 30.60 | 6m: 39.00 | 18m: 44.00 | 9y: 47.50 | 14y: 47.50</td></tr>
<tr><td>16</td><td>M</td><td>-</td><td>0m: 33.00 | 14y: 51.00</td></tr>
</table>

17	M	-	0m: 34.00 ǀ 12m: 43.00 ǀ 7y: 47.50 ǀ 10y: 49.30
76	M	-	8y: 48.50 ǀ 10y: 50.00 ǀ 16y-27y: 54.00 ǀ >=40y: 54.00
87	F	-	0m: 33.00 ǀ 4y: 47.00 ǀ 14y: 50.10 ǀ 28y-39y: 52.50
239	F	-	0m: 32.50 ǀ 7y: 45.50 ǀ 9y: 46.00
61	F	-	0m: 30.00 ǀ 4m: 38.00 ǀ 18m: 42.00 ǀ 24m: 43.00 ǀ 12y+6m: 48.50
45	M	+	0m: 32.00 ǀ 12m: 45.00 ǀ 3y: 49.00 ǀ 16y-27y: 55.00
274	F	+	0m: 34.00 ǀ 24m: 45.00 ǀ 2y+6m: 46.00 ǀ 5y: 50.00 ǀ 7y: 51.00
75	M	-	0m: 30.00 ǀ 5y+6m: 45.50
98	M	-	0m: 30.50 ǀ 8m: 40.20 ǀ 18m: 42.50 ǀ 3y: 45.00 ǀ 16y-27y: 53.00
214	M	+	0m: 32.00 ǀ 18m: 47.50
280	F	+	0m: 32.00 ǀ 12m: 44.00 ǀ 24m: 44.50 ǀ 2y+6m: 45.50
213	F	-	8y: 44.20 ǀ 11y: 46.00 ǀ 13y: 47.00
95	M	+	13y: 53.50 ǀ 16y-27y: 60.00 ǀ >=40y: 60.00
137	F	-	0m: 32.00 ǀ 6m: 41.00 ǀ 18m: 43.70 ǀ 2y+6m: 45.50 ǀ 16y-27y: 51.00
215	M	-	0m: 32.50 ǀ 3m: 36.20 ǀ 9m: 40.00 ǀ 3y+6m: 43.50
51	F	+	0m: 33.50 ǀ 3y+6m: 49.00
34	F	-	0m: 32.00 ǀ 4m: 35.00 ǀ 18m: 41.00 ǀ 10y: 47.50
46	M	-	0m: 32.00 ǀ 18m: 42.50
85	M	-	0m: 34.00 ǀ 8y: 47.00 ǀ 28y-39y: 50.50
108	F	-	0m: 34.00 ǀ 3y: 47.00 ǀ 13y+6m: 51.00 ǀ 28y-39y: 53.50
89	F	-	0m: 30.70 ǀ 7m: 38.50 ǀ 18m: 42.50 ǀ 14y: 50.20 ǀ 16y-27y: 50.50

SI Table S3

Table S3. Missing data (--, n.ac., n.av.) have been filtered out dynamically. Deletion size is standardized and evaluated in Megabases (Mb), while *TRIO* status is modeled as *TRIO* loss of one allele. SE: Standard error; ^: non-significant; *: p<.05; **p<.01.

Dependent Variable	Predictor Variable	Coefficient (beta)	(SE)	t-statistic	p-value
**Sitting Age**	***TRIO* Loss**	**6.01**	**2.55**	**2.35**	**0.023 ***
Model (N = 50)	**Deletion Size (Mb)**	**-0.02**	**0.06**	**-0.44**	**0.659**
**Walking Age**	***TRIO* Loss**	**23.97**	**7.74**	**3.10**	**0.003 ****
Model (N = 46)	**Deletion Size (Mb)**	**-0.04**	**0.17**	**-0.24**	**0.813**
**Speech Age**	***TRIO* Loss**	**15.33**	**13.43**	**1.14**	**0.259 ^**
Model (N = 47)	**Deletion Size (Mb)**	**0.56**	**0.69**	**0.81**	**0.420**

SI Table S4

Table S4. Birth HC in full-term infants with a preserved (+) or deleted (-) *TRIO* gene.

Cohort	Valid Cases (N)	Mean Birth HC (cm)	Standard Deviation (SD)
TRIO+ (Preserved)	7	32.93	0.93
TRIO- (Deleted)	25	31.88	1.79

SI Table S5

Table S5. Sex-stratified comparison of developmental milestones under homogeneous genetic backgrounds. Descriptive milestones parameters (sitting alone, independent walking, and speech) tracked in months, stratified strictly by child sex (Females vs. Males) and isolated within clear genetic boundary definitions: *TRIO*+ (both alleles functional, total (n=12) and *TRIO*- (one copy lost, total (n=40). Probability values ((p)-values) define two-tailed Mann-Whitney U test calculations evaluating structural sexual dimorphisms.

Genetic Status	Milestone (Months)	Sex	Valid (N)	Missing / Censored (n)	Mean	SD	Median	Mann-Whitney U (p)-value
**TRIO+ Group**	**Sitting Age**	**Females**<br>**Males**	**6**<br>**6**	**0**<br>**0**	**9.50**<br>**10.33**	**2.07**<br>**4.23**	**10.0**<br>**11.0**	**p = 0.936**<br>**(n. s.)**
	**Walking Age**	**Females**<br>**Males**	**6**<br>**6**	**0**<br>**0**	**18.50**<br>**22.00**	**2.43**<br>**6.16**	**18.0**<br>**22.0**	**p = 0.297**<br>**(n. s.)**
	**Speech Age**	**Females**<br>**Males**	**5**<br>**6**	**1**<br>**0**	**14.80**<br>**16.00**	**3.96**<br>**4.10**	**14.0**<br>**15.5**	**p = 0.684**<br>**(n. s.)**
**TRIO– Group**	**Sitting Age**	**Females**<br>**Males**	**21**<br>**17**	**1**<br>**1**	**14.33**<br>**16.94**	**5.37**<br>**10.02**	**12.0**<br>**15.0**	**p = 0.443**<br>**(n. s.)**
	**Walking Age**	**Females**<br>**Males**	**21**<br>**15**	**1**<br>**3**	**38.86**<br>**50.07**	**16.273**<br>**31.52**	**36.0**<br>**48.0**	**p = 0.354**<br>**(n. s.)**
	**Speech Age**	**Females**<br>**Males**	**21**<br>**15**	**1**<br>**3**	**39.00**<br>**38.20**	**28.52**<br>**33.68**	**30.0**<br>**36.0**	**p = 0.923**<br>**(n. s.)**

SI Table S6

Table S6. Comparison of data about HC, sitting, walking and speech ages between the whole group of cases and a reduced group excluding cases with complex karyotypes (i.e. cases ID33, ID100, ID2, ID110, ID213, ID95, ID46). SD: Standard Deviation; ns: not significant.

Variable	Group	Mean	SD	t	p-value
**hc**	Whole<br>Reduced	31.58<br>31.54	1.87<br>1.78	0.122	0.903 (ns)
**sitting**	Whole<br>Reduced	13.85<br>13.79	7.29<br>7.37	0.046	0.963 (ns)
**walking**	Whole<br>Reduced	36.70<br>36.94	22.92<br>23.95	-0.050	0.961 (ns.)
**speech**	Whole<br>Reduced	31.56<br>32.27	27.49<br>28.20	-0.125	0.901 (ns)

SI Figure S2

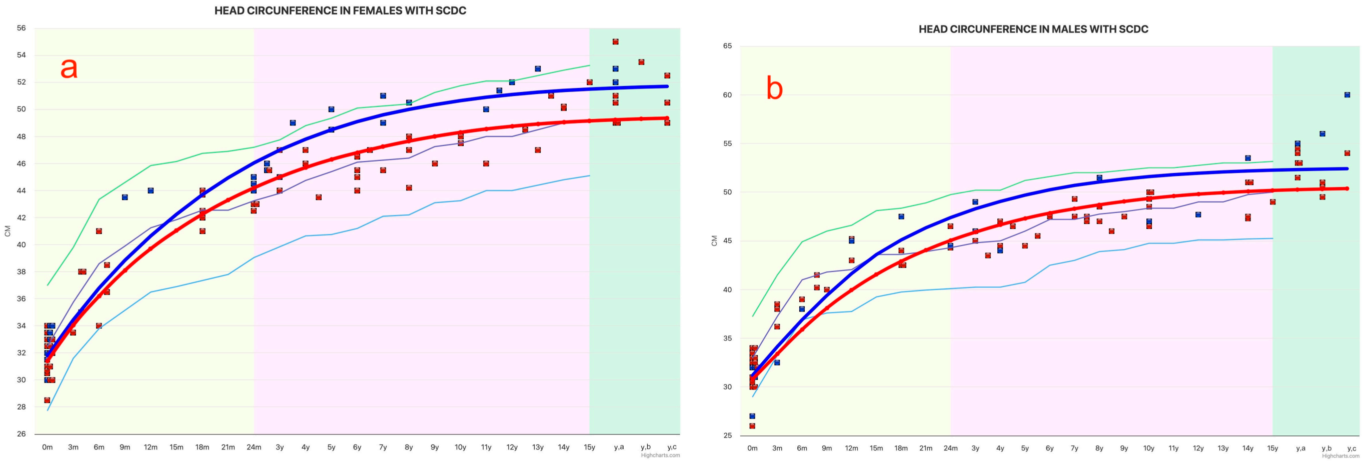

Figure S2. a: Distribution of all available HC measurements (n = 99) for females (29 patients) on normative curves. b: Distribution of all available HC measurements (n = 104) for males (28 patients). Multiple measurements for each individual at different ages are reported in SI Table S2. Gompertz curves are shown for patients with one *TRIO* allele (red) and for those with two *TRIO* alleles (blue); y,a; y,b; y,c, arefers to age groups as in Table S2.

SI Table S7

Table S7. Birth head circumference distribution across gestational age. Summary statistics expressing absolute neonatal head circumference (HC) in centimeters, stratified by gestational maturity at birth: =<37 completed weeks vs. =>38 completed weeks for the whole studied cohort (N=46) and analyzed separately by biological sex (26 females, 20 males). Probability values (p)-values define the output of two-tailed independent testing evaluating the physiological impact of intrauterine duration on syndromic microcephaly severity. SD: Standard deviation; *($p < 0.01$)

Cohort Group	Gestational age	Cases (N)	Mean Birth HC (cm)	(SD)	Group Comparison Significance (\(p\)-value)
Whole Group	=<37w<br>=>38w	16<br>30	30.76<br>32.22	1.83<br>1.57	Welch's t-test: (p = 0.007)*<br>Mann-Whitney U: (p = 0.012)
Females (\(F\))	=<37w<br>=>38w	9<br>17	30.74<br>32.12	1.95<br>1.52	Welch's t-test: (p = 0.065)<br>Mann-Whitney U: (p = 0.123)
Males (\(M\))	=<37w<br>=>38w	7<br>13	30.77<br>32.35	1.83<br>1.68	Welch's t-test: (p = 0.078)<br>Mann-Whitney U: (p = 0.052)

SI Table S8

Table S8. F: female; M: male; *TRIO*+: presence of two alleles; *TRIO*-: presence of one allele; FT1: females carrying one *TRIO* allele; FT2: females carrying two *TRIO* alleles ; MT1: males carrying one *TRIO* allele MT2: males carrying two *TRIO* alleles; >50°:above 50°centile; <50°:<below 50° centile; ^: not significant at p<.05, after Yates correction; * significant at p <.05; ** significant at p< .01; =>38w: birth at or after the 38th week of pregnancy; =<37w: birth at or before the 38th week of pregnancy; n.a.: statistical test not applicable because small number of cases.

M/F and TRIO+/TRIO-	χ: .042; p: .836 ^	TRIO+ (=<37w) [mean hc F vs M]	t: 0.365; p: .738^
M/F and hc>50°/hc<50°	χ: 0.19; p; .660^	TRIO+ (=>38w) [mean hc F vs M]	n.a.
TRIO+ [F/M vs =>38w/=<37w]	χ: 1.07; p: .300^	TRIO- (=<37w) [mean hc F vs M]	t: -0.079; p: .938^
TRIO- [F/M vs =>38w/=<37w]	χ: $1x10^{-4}$; p: .991^	TRIO- (=>38w) [mean hc F vs M]	t: 0.208; p: .837^
FT1/FT2 vs >50°/<50°	χ: 2.68; p: .101^	TRIO+ (=<37w) [mean deletion size F vs M]	t: -0.031; p: .976^
MT1/MT2 vs >50°/<50°	χ: 0.10; p: .751^	TRIO+ (=>38w) [mean deletion size F vs M]	n.a.
FT1/FT2 vs =>38w/=<37w	χ: 0.02; p: .881^	TRIO- (=<37w) [mean deletion size F vs M]	t: -0.464; p: .651^
MT1/MT2 vs =>38w/=<37w	χ: 0.32; p: .570^	TRIO- (=>38w) [mean deletion size F vs M]	t: -0.795; p: .436^

FT1 (hc) [mean =>38w vs =<37w]	t: 2.701; p: .015*	MT1 (hc) [=>38w vs =<37w]	t: -1.315; p .213^
FT2 (hc) [mean =>38w vs =<37w]	t: 3.031; p: .023*	MT2 (hc) [=>38w vs =<37w]	n.a.
FT1 (deletion size) [=>38w vs =<37w]	t: 0.271; p: .789^	MT1 (deletion size) [=>38w vs =<37w]	t: 0.027; p .978^
FT2 (deletion size) [=>38w vs =<37w]	t: 0.271; p: .794^	MT2 (deletion size) [=>38w vs =<37w]	n.a.
MT1/FT1 (hc) [mean =>38w vs =<37w]	t: -2.772; p: .009**	deletion size [M vs F]	t: -1.209; p: .232^
MT2/FT2 (hc) [mean =>38w vs =<37w]	t: -3.096; p: .011*	M/F (hc) [all weeks]	t: 1.098; p: .277^

SI Table S9

Table S9. Correlation analysis developmental milestones vs deletion size. hc: head circumference; deletion size: deletion size; r: Pearson's Correlation Coefficient; ^: non-significant; *: significant <.05; **: significant, < .01 or lower; §: sample size too small to calculate r.

	**hc/deletion size**	**sitting age/deletion size**	**walking age/deletion size**	**speech age/deletion size**
FT-	r: -0.145; p: .567^	r: -0.237; p: .328^	r: 0.361; p: .141^	r: 0.225; p: .368^
FT+	r: -0.101; p: .813^	r: 0.598; p: .088^	r: -0.047; p: .912^	r: 0.145; p: .755^
FT+/FT-	r: -0.261; p: .188^	r: 0.181; p: .366^	r: 0.607; p: .000987**	r: 0.438; p: .028*
MT-	r: -0.129; p: .660^	r: -0.253; p: .311^	r: 0.292; p: .238^	r: 0.187; p: .457^
MT+	§	r: 0.722; p: .168^	r: 0.919; p: .0268*	§
MT+/MT-	r: 0.121; p: .632^	r: -0.016; p: .942^	r: 0.496; p: .0158*	r: 0.317; p: .149^
FT+/FT- #MT+/MT-	r: -0.099; p: .517^	r: 0.119; p: .407^	r: 0.538; p: .000065**	r: 0.380; p: .008**
